# Farmer–veterinarian interaction as multi-level situated learning: Negotiating health, risk, and responsibility in intensive pig farming — a scoping review

**DOI:** 10.1016/j.onehlt.2026.101401

**Published:** 2026-04-01

**Authors:** Rolien Willmes, Jasper de Vries, Bob Mulder, P. Marijn Poortvliet, Laurens Klerkx

**Affiliations:** a Strategic Communication Group, Wageningen University, Hollandseweg 1, 6706 KN Wageningen, the Netherlands; b Land Use Planning Group, Wageningen University, Droevendaalsesteeg 2, 6708 PB Wageningen, the Netherlands; c Cultural Geography Group, Wageningen University, Droevendaalsesteeg 2, 6708 PB Wageningen, the Netherlands; d Departamento de Economía Agraria, Facultad de Ciencias Agrarias, Universidad de Talca, Campus Lircay, Talca, Chile; e Knowledge, Technology and Innovation Group, Wageningen University, Hollandseweg 1, 6706 KN Wageningen, the Netherlands; f Center for Advancing Agri-Food System Transformation (CA2FST), Santiago, Chile

**Keywords:** Veterinarian–farmer interaction, Health governance, Intensive farming, Pig farming, Antimicrobial use, Socio-ecological systems

## Abstract

Farmer–veterinarian interaction is central to how responsibility for animal health and antimicrobial use is enacted. Especially in intensive pig systems, where high animal density, economic pressures, and stringent biosecurity requirements elevate both zoonotic risks and public health concerns. Yet, empirical research on these interactions remains limited as these are often embedded in broader animal health studies and disproportionately focused on dairy contexts.

This scoping review synthesizes 40 empirical studies on farmer–veterinarian interaction in intensive pig farming, applying PRISMA-ScR methodology and a four-level socio-ecological framework encompassing individual, interpersonal, interprofessional, and systemic influences. Eligible sources included all studies including relevant empirical insights published until July 2023, with no geographical restrictions, retrieved from Scopus, Web of Science, Agricola, and CAB Abstracts.

Findings indicate that veterinarians in intensive pig farming are increasingly positioned as strategic partners in herd health management. Their influence depends not only on technical expertise but also on trust, embeddedness in local practice, and their role in enabling ongoing learning in complex farm environments. However, tensions persist. Farmers may turn to peer networks over professional advice in times of stress, especially under economic pressure, and divergent views and priorities concerning animal health management, welfare, and antimicrobial use can complicate interaction. Structural pressures—such as short-term economic incentives, market constraints, and the dual commercial-regulatory role of veterinarians— further limit joint decision-making capacity. Although zoonotic risk and antimicrobial resistance dominate macro-level policy discourse, these concerns did not emerge as central themes in the empirical literature on everyday farmer–veterinarian interaction.

By adopting a multi-level lens, this review exposes the layered complexity of human–animal health governance in industrial livestock systems. The synthesis offers a systems-aware perspective on how contextually situated relationships shape management behaviours, health and biosecurity outcomes. These insights support more effective policy design and cross-sectoral collaboration at the livestock–public health interface for resilient livestock farming.

## 1 Introduction

Global meat production has more than tripled in the past decades, rising from around 100 million metric tons in 1980 to over 360 in 2023 [[1], [2]]. This expansion has been driven largely by the rise of industrial livestock systems, especially in pigs and poultry, which deliver efficiency and output but also heighten concerns about antimicrobial resistance (AMR), zoonotic spillover, animal welfare, and environmental sustainability [[1], [3]]. With farm consolidation and intensification expected to continue [4], there is an immediate challenge to enhance the resilience of livestock farming systems by strengthening how human actors enact responsibility for the health and lives of animals, while broader debates about their future direction continue.

Veterinarians are central actors in this transition. Their roles have broadened from treating individual animals to include herd health management, disease prevention, biosecurity, and overall risk mitigation—elements that underpin resilient livestock farming practices [[5], [6], [7], [8]]. Especially in intensive systems, effective collaboration between farmers and veterinarians is essential for developing tailored, context-specific solutions that balance productivity with animal health, welfare, and sustainability [[9], [10]].

Studying the dynamics of farmer–veterinarian interaction is particularly relevant in pig farming—an industry characterized by high herd densities, confinement, and extensive antimicrobial use [[11], [12], [13], [14]]. These features create unique challenges and environmental risks, positioning pig systems at the forefront of One Health concerns—where animal health, human health, and ecosystems are interconnected [15]. Understanding how relationships develop, how trust is built, and how systemic, social and communicative factors influence decision-making is crucial for supporting forms of herd health management in which actors enact and negotiate responsibility for animal health, antimicrobial use, and broader sustainability outcomes. We approach these interactions as processes of negotiation, in which farmers and veterinarians align perspectives, knowledge, and responsibilities to arrive at provisional trade-offs regarding animal health and its management [[16], [17], [18]].

Despite their importance, research on farmer–veterinarian interactions in pig farming remains limited and fragmented. Pig farming research has primarily focused on health outcomes like antimicrobial use and disease prevalence, with less attention paid to the social and relational processes that underpin effective collaboration [[19], [20], [21]]. In the broader domain, studies in dairy farming have examined these relationships across multiple social levels—such as individual expertise, relational trust, and systemic influences. These insights are however rarely integrated into a comprehensive framework. This lack of integration hinders efforts to develop strategies that strengthen veterinarian–farmer partnerships and better understand how responsibility for animal health and sustainability is enacted in practice.

To address this gap, this review systematically maps and synthesizes empirical research on farmer–veterinarian interaction in intensive pig farming. Drawing on socio-ecological and systems-oriented perspectives, we develop a four-level analytical framework—spanning individual, interpersonal, interprofessional, and systemic influences—to enable a holistic account of farmer–vet interactions and their broader contexts [[22], [23], [24]]. While elements of these levels have been examined separately in dairy research, no study has yet applied such an integrative lens to livestock farming. Applying this framework here not only advances academic understanding of agricultural change but also generates insights directly relevant to ongoing policy and practice debates at the intersection of animal, human, and environmental health [25]. Analysing these dimensions in isolation risks overlooking how dynamics at one level condition or constrain action at another. For example, how interpersonal trust is shaped by systemic incentives or how individual expertise depends on interprofessional networks. Integrating these levels therefore enables a more coherent account of how farmer–veterinarian interaction unfolds in practice.

The objective of this scoping review was thus to systematically map and synthesize empirical research on farmer–veterinarian interactions in intensive pig farming. Specifically, we asked: (1) What empirical insights exist on how these interactions are studied? (2) Which factors across individual, interpersonal, interprofessional, and systemic levels shape these interactions? (3) What knowledge gaps remain for future research and practice?

A scoping review methodology was chosen rather than a systematic review because the literature on farmer–veterinarian interactions in pig farming is dispersed across disciplines, heterogeneous in study design, and often embedded within broader research on animal health. Scoping reviews are particularly suited to mapping such fragmented evidence bases, identifying conceptual frameworks, and highlighting research gaps. Unlike systematic reviews, they do not aim to aggregate effect sizes or assess intervention effectiveness, but rather to chart the breadth, nature, and conceptual structure of an emerging field.

## 2 Methods

### 2.1 Conceptualizing farmer–veterinarian interaction as a multi-level situated learning process

Veterinary advisory work is increasingly viewed not as a one-way transmission of knowledge but as an interactive and context-sensitive process. Scholars argue that the effectiveness of veterinary recommendations hinges on their alignment with the specific conditions of individual farms [[5], [26], [27]]. While veterinarians may prioritize clinically oriented or preventive herd health goals, farmers must also weigh practical constraints such as financial constraints, labour availability, and infrastructure. For instance, the feasibility of implementing hygiene protocols may be limited by farm layout or staffing, especially during weekends or night shifts [28]. In this light, farmer–veterinarian interaction can be understood as a process of negotiation, in which actors bring together different forms of knowledge, interests, and responsibilities to define problems and possible courses of action [[16], [17], [18]]. These negotiations are shaped by asymmetries in expertise and dependency, and lead to provisional trade-offs between what is epidemiologically desirable, economically feasible, and practically manageable.

As these interactions unfold over time within specific farm contexts, they can be understood as a process of situated learning, in which knowledge is co-produced through shared practices and the ongoing negotiation of roles and relationships ([[29], [30]]; see also [[31], [32], [33]]). Understanding such learning processes in practice requires attention to the multiple contexts through which meanings are negotiated and actions become feasible—for example; how decisions about treatment versus prevention are shaped not only by individual knowledge; but also by trust in the veterinarian; input from other advisors; and regulatory or market pressures. From this perspective; negotiation and learning are shaped by interdependent factors operating across multiple socio-ecological levels; as reflected in existing work on farmer learning and advisory systems (e.g. [[29], [34]]). Analysing farmer–veterinarian interaction therefore requires an approach that captures how these processes are embedded within and structured by these interconnected levels.

**Fig. 1:**
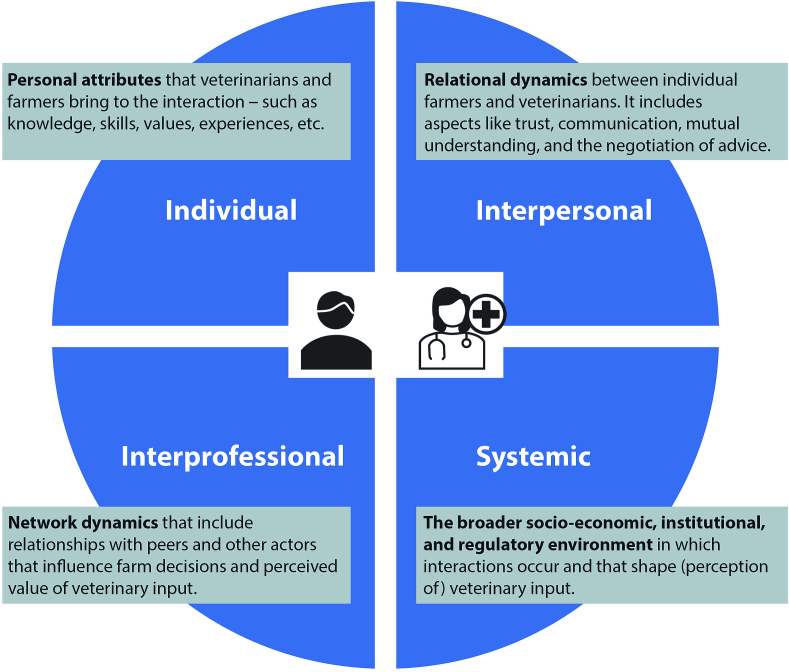
Farmer–vet interaction as a situated learning process on different socio-ecological levels. Visual presentation of the four-level analytical lens for analysing farmer-vet interaction as a layered situated learning process (created by authors).

Building on this multi-level perspective, our framework (see Fig. 1) distinguishes between the following levels:

• *Individual level:* This level concerns the personal attributes—such as knowledge, skills, values, experiences, etc.—that veterinarians and farmers bring to the interaction. Veterinary involvement is for example studied on this level in studies by Proctor et al. [28] or Ingram [35].
• *Interpersonal level:* This level focuses on the relational dynamics between individual farmers and veterinarians. It includes aspects like trust, communication, mutual understanding, and the negotiation of advice. This interpersonal perspective on veterinary involvement can for example be found in studies of Hilkens et al. [36] or Hamilton [27].
• *Interprofessional level:* At this level, attention shifts to the broader network of professionals involved in livestock management. Farmer-veterinarian interactions are influenced by relationships with peers (other vets and other farmers) and other actors such as nutritionists, breeding advisors, and pharmaceutical representatives. This contextual level to veterinary involvement is for example described by Phillipson et al. [37] or Smith and Hollis [38].
• *Systemic level:* This level encompasses the broader socio-economic, institutional, and regulatory environment in which interactions occur. This level of context to farmer-vet interaction is addressed in studies like: Sutherland and Labarthe [39] or Hurd [40].

### 2.2 Design

Given the breadth of the objectives and diversity of evidence, a scoping review was selected as the most suitable method. The review followed the PRISMA-ScR checklist [41] (see Appendix A) and conceptualized farmer–veterinarian interaction as a multi-layered process influenced by individual, interpersonal, interprofessional, and systemic contexts as described above.

### 2.3 Search methods

A comprehensive search was carried out in Scopus, Web of Science, Agricola, and CAB Abstracts to capture both veterinary and agricultural literature. The search strategy was iteratively developed to include terms in three areas:

• *Veterinarian involvement* (e.g., “veterinary practitioner,” “vet,” “veterinary consultation”).
• *Farmer–veterinarian interaction* (e.g., “communication,” “trust,” “collaboration,” “advice”).
• *Pig farming* (e.g., “pig,” “sow,” “swine,” “porcine”).

Boolean operators (AND/OR) were used to combine terms and ensure coverage of the diverse terminology in the field. This resulted in the following search string for use in Web of Science and Scopus:

((“veterinary practitioner” OR “veterinary advice” OR “veterinary practice” OR “veterinary consultation” OR “veterinary surgeon” OR veterinarian OR “veterinary service” OR vet) AND (communication OR communicate OR interaction OR vet-farmer OR farmer-vet OR veterinarian-farmer OR farmer-veterinarian OR collaboration OR co-production OR co produce OR relationship OR “relationship factor” OR “cultural script” OR “behavior change” OR “behaviour change” OR advice OR advisory OR coach OR coaching OR counseling OR consult OR consultation OR extension OR “knowledge transfer” OR trust OR management OR program OR protocol) AND (pig OR piglet OR sow OR weaner OR “fattening pig” OR hog OR swine OR porcine))

An adapted version of this string was used for Ovid databases, as these work with different search operators (see Appendix A).

### 2.4 Eligibility criteria

Studies were included if they examined farmer–veterinarian interactions in intensive pig farming, focusing on communication, collaboration, and impacts on pig health, welfare, and farm management. Both peer-reviewed and grey literature were considered to capture practitioner and industry perspectives [[42], [43]] Sargeant and O'Connor [44]. Exclusion criteria covered studies on other livestock, non-English publications, and work lacking sufficient relevance or data. The review was conducted in July 2023. Inclusion criteria allowed studies from any location, published from 1980 onwards.

### 2.5 Search outcomes

**Fig. 2:**
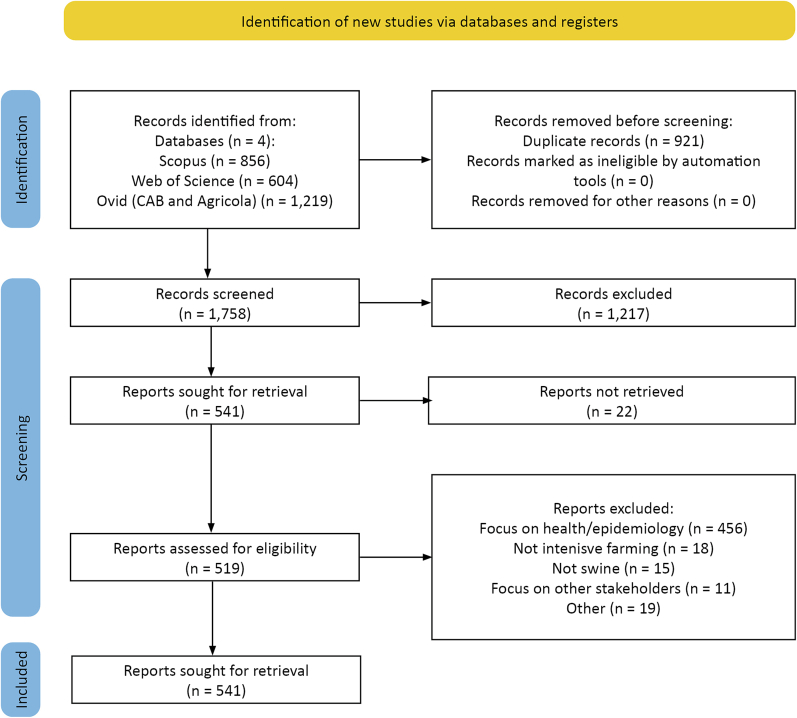
PRISMA flow diagram.

Screening was performed in Rayyan [45]. Two reviewers independently assessed titles and abstracts, resolving disagreements with a third. The search yielded 2.679 records: 1.219 from Ovid databases (Agricola and CAB abstracts), 856 from Scopus and 604 from Web of Science. After duplicate removal, 1758 records were screened at title and abstract level, of which 541 were retained for full-text assessment. Applying the predefined inclusion and exclusion criteria (Section 2.4) resulted in 40 studies meeting all criteria. The full selection process is presented in the PRISMA flow diagram below (Fig. 2).

### 2.6 Data extraction and synthesis

Data were extracted using a pilot-tested protocol, covering study characteristics and findings on farmer–veterinarian dynamics. The reflexive thematic analysis followed four steps

For each included study, the following data items were extracted: bibliographic information (author, year, country), study design and methodology, study population and sample size, context (production system, farm characteristics), outcomes reported (e.g., health management, antimicrobial use, welfare impacts), and key findings relevant to our four-level socio-ecological framework.

1. *Study selection:* 40 studies yielded 196 discrete empirical findings.
2. *Categorization:* Findings assigned to the four socio-ecological levels.
3. *Within-level analysis:* Recurring patterns identified at each level.
4. *Cross-level analysis:* Overarching themes cutting across levels were synthesized.

### 2.7 Quality appraisal

Although formal quality appraisal is not required for scoping reviews, we assessed methodological rigor to contextualize the evidence. Studies were evaluated against basic criteria including clarity of research questions, appropriateness of methods, sample adequacy, and transparency of reporting. Most employed solid quantitative, qualitative, or mixed-method designs, though common limitations included small samples, descriptive analyses, and reliance on self-reported data. This appraisal informed the synthesis but was not used to exclude studies.

## 3 Results

In this section, we present the findings from the analysis of the 40 included studies. We begin by outlining the characteristics, including temporal and topographical aspects, linking to the extraction table for details (see Appendix A). Following this, we provide an overview of the within-level factors identified, categorizing them according to our conceptual framework, which distinguishes four contextual levels: individual, interpersonal, interprofessional, and systemic. We then move to a more extensive discussion of the overarching themes that emerged from the across-level dynamics, highlighting how factors at different levels interact.

### 3.1 Study characteristics

**Fig. 3:**
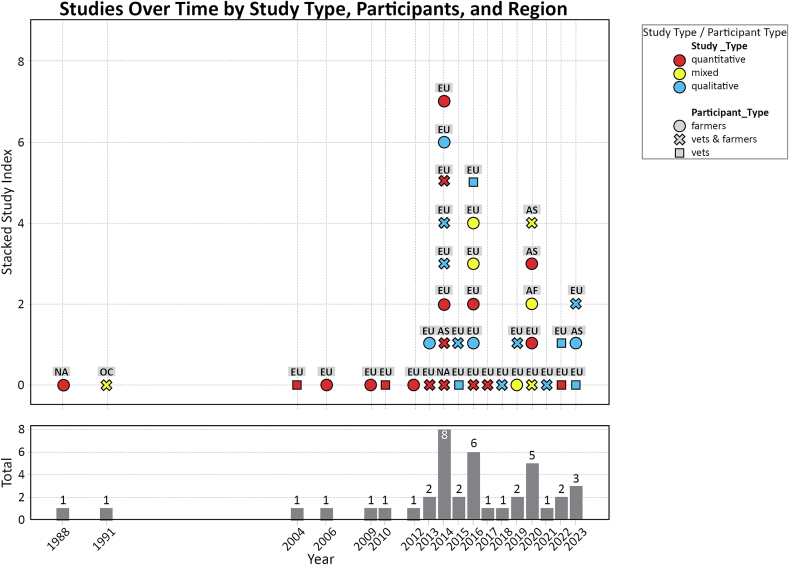
Visual overview of the characteristics of included studies.

This review synthesizes findings from 40 empirical studies [link to extraction table] on farmer–veterinarian interactions within intensive pig farming. A visual overview of the study specifications can be found in Fig. 3, indicating type of study (quantitative or qualitative) and the temporal and geographical distribution of studies.

### 3.2 Overview within level factors

**Fig. 4:**
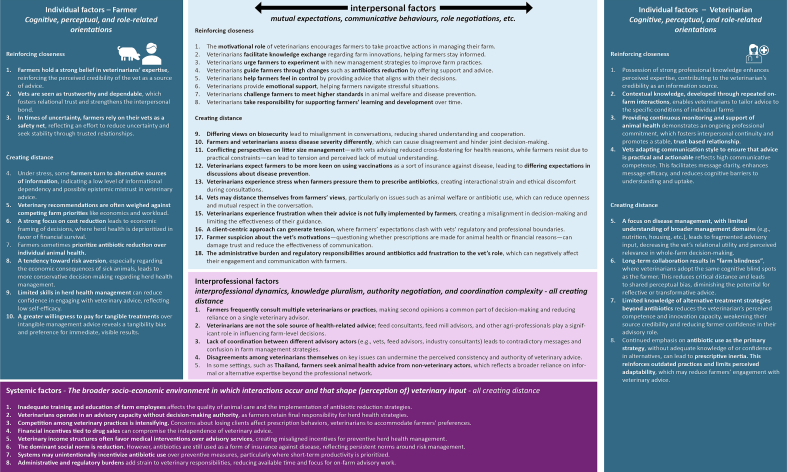
Factors influencing farmer–vet interaction at different socio-ecological levels, as identified from the selected studies

A factor-based analysis was conducted within each socio-ecological level to identify recurring and influential factors that shape farmer–veterinarian relationships in the context of intensive pig production. This level-specific analysis allows for a granular understanding of the factors influencing farmer-vet interaction at each level, as identified from the selected studies. The results of this analysis are presented in Fig. 4, organized as factors sorted by socio-ecological level.

### 3.3 Across-level analysis for finding overarching themes

After identifying factors within each contextual level (Fig. 4), a second thematic analysis explored cross-level patterns. The aim was to identify overarching themes that emerged across levels. To show these links, factors are indicated by socio-ecological level (individual, interpersonal, interprofessional, systemic) and number (e.g., individual factor 1 V, with V = vet, F = farmer).

#### 3.3.1 Overarching Theme 1: Foundations of the strategic partnership between farmer and veterinarian

Veterinarians are positioned not only as service providers but as strategic partners in farm management. They influence both daily operations and broader health strategies. We found three subthemes in the literature that underpin this role.

##### 3.3.1.1 Subtheme 1. The vet as credible expert

Studies highlight veterinarians as trusted, credible sources of information, ranking above other advisors [[46], [47], [48], [49], [50], [51], [52], [53]]. One farmer noted: “Mainly my vet. I have got confidence in my vet. So that's who I listen to first of all” [46]. Research in Belgium [54] and the UK [46] underscores the value of expertise (Individual factor 1V). Farmers expect vets to act as experts (Individual factor 1F); especially in diagnosis and disease management. As Maes et al. [54] state: “Strong professional knowledge is the best guarantee for veterinary business support that will continue to provide significant added value to the pig farming sector in the future.”

##### 3.3.1.2 Subtheme 2. Experiential knowledge creates trust

Trust, credibility, and prior experience shape farmers' reliance on advice [55]. Vets are often viewed as more credible than other advisors (Individual factor 2F) [[46], [50], [55], [56]]. Farmers value contextualized knowledge (Individual factor 2V)—an understanding of local disease conditions and practices, built through regular farm visits. UK studies particularly emphasize this expectation [[46], [55]]. As Garforth et al. [55] note, “farmers considered his or her veterinarian as a ‘fieldperson’, who is constantly visiting many other pig units and therefore had the knowledge of the disease situation in the country/area and the experience to prevent and control the situation.”

##### 3.3.1.3 Subtheme 3. Vets support knowledge exchange and innovation

Veterinarians also play a motivational role, encouraging farmers (Interpersonal factor 1) to adopt new practices [50]. French studies highlight vets' role in knowledge exchange (Interpersonal factor 2) and experimentation (Interpersonal factor 3) [[19], [57]]. As one farmer explained: “I generally tend to trust my vet for everything to do with health... we've known how to raise pigs for years... It's all old hat” [57]. Reflecting this dynamic; Joly et al. [19] notes that “experiments frequently stem from external recommendations by veterinarian practitioners or other key actors of ‘animal health’ farmer organizations.”

UK-based research further shows that farmers often view veterinarians as coaches, particularly during transitions such as reducing antibiotic use (Interpersonal factor 4). As Golding et al. [50] observe; “farmers mostly felt vets were the experts who could provide them with support and motivation to reduce antimicrobial use on-farm.” Several studies also emphasize that veterinarians can enhance farmers' sense of control (Interpersonal factor 5) over the outcomes of management changes by offering consistent support and monitoring (Individual factor 3V). As Alarcon et al. [46] note, “having a measurable outcome contributed to farmers' feeling of control of the situation.”

Beyond technical support, some studies describe veterinarians as offering emotional support (Interpersonal factor 6), particularly in regulatory or high-pressure contexts. A German study illustrates how veterinarians provide empathy while also encouraging higher welfare standards (Interpersonal factor 7) [58]. One pig vet commented: “There's so much coming over him […]. In the end I do have a bond with the farmers... it is close to my heart if they are not doing well.” At the same time, however, some veterinarians distanced themselves from farmers (Interpersonal factor 14), emphasizing a more directive approach: “This is the pig, that's how it is made... adapt your system to the pig the way it is.”

Veterinarians are also portrayed as taking responsibility for farmers' learning and development (Interpersonal factor 8), often by making it actionable (Individual factor 4V). One veterinarian described tailoring advice: “As far as they are concerned, the metering pump may as well be a nuclear power plant” [57]. Another emphasized: “I want them to understand why I say this or that” [57]. In doing so; veterinarians become sources of reassurance and confidence in uncertain situations (Individual factor 3F) [46].

#### 3.3.2 Overarching Theme 2: Misalignments and negotiated trade-offs challenging the strategic partnership

Misalignments complicate farmer–vet collaboration. Across the literature, these misalignments frequently reflect differing trade-offs across value dimensions rather than simple disagreement. Farmers and veterinarians weigh economic viability, animal welfare, labor constraints, regulatory expectations, and long-term herd health differently. The tensions observed can thus be understood as situated negotiations of competing priorities rather than failures of knowledge alone. Four subthemes emerge from the literature.

##### 3.3.2.1 Subtheme 1. Turning to peers for advice

Farmers often seek peer advice under stress (Individual factor 4F), perceiving peers as better attuned to practical realities. Under pressure, they may revert to traditional practices instead of veterinary recommendations [[19], [46], [59]]. One farmer said: “You check with your vet… but I also belong to a working group… Some are more pioneering… Others… wait-and-see… I'm more in the second category” [19]. Cost is a common reason for second opinions: “Just because the vet says do it; we don't just jump… we enquire” [46]. Peer influence can reinforce or undermine veterinary advice: “We were just again back against the wall… and when I heard this from my Irish friend… I bought the stuff” [46]. Collective structures such as French CETA groups or Dutch farmer study groups also rank just behind vet advice [[19], [53]].

##### 3.3.2.2 Subtheme 2. Different understandings of animal health

Vets emphasize welfare and ideal outcomes, while farmers weigh these against cost and workload (Individual factor 5F). These differences illustrate trade-offs between welfare optimization and economic or organizational feasibility at farm level. Economic pressure may drive reliance on antibiotics instead of preventive measures [60] or incomplete records [61]. Research from Hungary further highlights how economic restructuring can affect herd health management (Individual factor 6F). As Bíró et al. [62] report; “Numerous new owners appeared in pig-breeding; often without any background knowledge on animal husbandry. These managers often try to follow a minimum cost strategy… [and] often neglect the veterinary considerations.” This illustrates how cost-cutting can override professional veterinary input in herd health decisions.

Farmers' reluctance to use antibiotics sometimes diverges from vets (Individual factor 7F). Vets for example describe “grey zones” after feed changes (ZnO reduction): “I see some pigs… perhaps they needed treatment. But mortality is unchanged… so it's difficult to argue against [the farmer's approach]” [63]. Conversely; vets sometimes insist: “To some; I sometimes have to say: ‘Listen… we simply need to carry out some treatments here’” [63]. Divergence also appears in biosecurity (Interpersonal factor 9); pain management (Interpersonal factor 10); and piglet care (Interpersonal factor 11) [[20], [64], [65]], although the dynamics surrounding antibiotic use stand out in the literature due to their regulatory intensity and entrenched prescribing cultures.

##### 3.3.2.3 Subtheme 3. Assessing and handling risk differently

A range of studies report on how farmers approach veterinary decision-making, often describing immediate, control-oriented strategies that can place pressure on vets to prescribe preventively. Here, trade-offs emerge between short-term risk mitigation and longer-term antimicrobial stewardship or preventive investment. For instance, vets note farmers would vaccinate more if allowed to do it themselves [66]. Antibiotic reduction highlights divergences: vets are more optimistic than farmers [67]. Pressure also causes strain: “There are 75% of rational clients… and 25% who simply insist [on antibiotics]” [61]. Legal fears reinforce antibiotic use: “You're laying yourself open to litigation if you don't use them” [61]. These patterns reflect elements of risk aversion; particularly where farmers prioritize avoiding immediate economic losses or legal repercussions over longer-term preventive strategies; potentially contributing to inertia in antimicrobial practices. Skill gaps (Individual factor 9F) in antibiotic need; pain recognition; and animal care—along with training deficiencies (Systemic factor 1)—shape outcomes [[63], [64], [68]]. Some vets distance themselves from farmers over antibiotics and welfare (Interpersonal factor 14) [61]; though shifting social norms encourage restraint [63].

##### 3.3.2.4 Subtheme 4. Balancing priorities

Farmers make final decisions (Systemic factor 2), limiting veterinary influence. This reflects structural trade-offs between advisory input and farm-level autonomy, as well as between economic survival and preventive ideals. This can frustrate vets (Interpersonal factor 15), though many still prescribe when animals suffer. Farmers value vets who consider economics (Individual factor 5 V) [46]. Opinions differ on whether vets should advise on feed/housing [60]. Concerns also arise around gaps in economics; environment; and agribusiness knowledge [[69], [70]]. Many vets adapt communication and engage in farmer education (Interpersonal factor 8) [[50], [57]].

#### 3.3.3 Overarching Theme 3: Challenges to being ‘the perfect vet’

Veterinarians are often expected to be ideal advisors, but this is unrealistic, as reflected in five subthemes.

##### 3.3.3.1 Subtheme. Farm blindness

Close ties can reduce objectivity (Individual factor 6V). “Farm blindness” occurs when long relationships cause vets to become so familiar with the farmer and the particular farm that they overlook the same issues [70].

##### 3.3.3.2 Subtheme 2. Financial dependence

Veterinarians' reliance on farmer income constrains how directive they can be (Interpersonal factor 16). “I have an enormous dependency… he pays my bill” [58]. Shrinking markets and competition (Systemic factor 3) add pressure; especially in the UK/Ireland where farmers often use multiple vets (Interprofessional factor 1). Prescribing is also influenced by convenience [68]. Vets report client pressure: “The client won't accept… They want you to come up with the right answer straight away” [61].

##### 3.3.3.3 Subtheme 3. Commercial interests

Drug sales contribute up to 43% of income [54] (Systemic factor 4). Though most farmers trust vets; perceptions of financial incentives persist (Interpersonal factor 17). Some propose separating income from drug sales; but farmers prefer convenience [21]. Alternative payment models (Systemic factor 5) encourage preventive strategies [[59], [66]]. Roles also vary (herd vets, feed mill vets, pharma-affiliated vets) [70].

##### 3.3.3.4 Subtheme 4. Curative medicine prevails

Studies also highlight structural and habitual factors that reinforce reliance on antibiotics in veterinary practice. Entrenched habits and systemic constraints sustain antibiotic reliance (Systemic factor 6). Limited vet knowledge of alternatives (Individual factors 7V & 8V) reinforces pharmaceutical focus [19]. Preventive measures like charcoal in Denmark show potential; but vets still “think treatment = antibiotics” [63]. Payment structures also hinder advisory work; as farmers prefer paying for treatments (Individual factor 10F). Some vets call for paid advice but fear losing clients (Interpersonal factor 16). Regulatory burdens add complexity: “When I started… two pieces of paper… Today I make forty-two” [63].

##### 3.3.3.5 Subtheme 5. Conflicting guidance

Farmers often receive advice from multiple sources (Interprofessional factor 2), including other veterinarians, nutritionists, breeding advisors, feed consultants, and pharmaceutical representatives. Poor communication sidelines vets: “Farmers often preferred to call an advisor” [49]. Inconsistent advice among vets (Interprofessional factor 4) or reliance on informal/unqualified contacts (Interprofessional factor 5) further complicate decisions [71].

## 4 Discussion

The aim of this review was to systematically map and synthesize empirical research on farmer–veterinarian interactions in intensive pig farming, identifying factors shaping these interactions across individual, interpersonal, interprofessional, and systemic levels. By mapping findings with this multi-level framework, the review offers a conceptual lens for understanding the complexity of farmer–veterinarian interaction.

### 4.1 Farmer–vet interaction in intensive pig farming

The analysis shows that farmer–veterinarian interactions in intensive pig production are shaped by influences across all four socio-ecological levels: individual, interpersonal, interprofessional, and systemic. Three overarching themes—(1) foundations of partnership, (2) misalignments, and (3) pressures shaping the “perfect vet”—underscore the complexity of these relationships.

Theme 1 highlighted the foundations of the farmer–vet partnership, showing that vets are regarded as credible experts (Individual factors 1V, 1F) whose contextual knowledge (Individual factor 2V) and role in knowledge exchange (Interpersonal factor 2) make them indispensable partners. Moreover, veterinarians are consistently ranked as farmers' most trusted advisors [[46], [47], [50]].

Theme 2 however, emphasized misalignments in how farmers and vets interpret health, risk, and economic trade-offs. As seen in subtheme 1, farmers often turn to peers (Individual factor 4F) when under stress, sometimes prioritizing experiential or cost-oriented advice over veterinary recommendations [[19], [46]]. It was discussed that differences in defining animal health (subtheme 2)—for instance, prioritizing cost or labor over welfare—can limit the uptake of veterinary advice [[61], [68]]. Furthermore, differing approaches to risk management (subtheme 3)—such as immediate, control-oriented farmer strategies versus vets' preventive outlooks [[66], [67]]—create pressure points in collaboration. Also, farmers' decision-making authority (subtheme 4) can frustrate vets, especially when economic constraints drive reliance on antibiotics rather than preventive measures [[62], [64]]. These dynamics suggest that risk aversion—especially in the face of economic uncertainty and potential disease loss—reinforces conservative decision-making and contributes to the persistence of antibiotic-centered routines. Collectively, these patterns indicate that farmer–veterinarian interaction is structured by recurring negotiations of trade-offs across economic, welfare, temporal, and risk-related considerations.

Theme 3 illustrated the pressures shaping the “perfect vet.” It was described that “farm blindness” (subtheme 1) due to long-term relationships can undermine objectivity [70]; as can financial dependence of vets on farmer clients (subtheme 2; Interpersonal factor 16) and the role of drug sales in shaping veterinary practice and farmer perceptions [[21], [54]]. These findings suggest that responsibility cannot be assumed to reside inherently in either professional role, but is shaped by structural incentives and commercial arrangements that condition advisory practice. Furthermore, the persistence of curative over preventive medicine (subtheme 4), reflected entrenched habits (Systemic factor 6) and knowledge gaps (Individual factors 7V & 8V) [[19], [63]]. Finally, conflicting guidance from multiple advisors—including nutritionists, feed consultants, pharmaceutical representatives, and other veterinarians (subtheme 5; Interprofessional factors 2, 4, 5)—undermines coherence in advisory practice. [[49], [71]].

Interestingly, although zoonotic risk features prominently in policy and public health discourse and framed the broader context of this review, it did not emerge as a central theme in the empirical literature on farmer–veterinarian interaction. This suggests a possible disconnect between macro-level One Health narratives and the issues that structure everyday advisory practice, where antimicrobial use, economic viability, and herd-level management concerns appear more salient.

### 4.2 Farmer–vet interaction as situated learning

This review demonstrates that farmer–vet interactions are embedded in interdependent contexts, as illustrated by the multi-level influences identified in 4.1. The key conceptual contribution of this study therefore is to frame farmer-vet interaction as situated learning across four socio-ecological levels. Rather than one-way advice transfer, knowledge and decisions are co-constructed through interaction. This aligns with systems thinking and Agricultural Knowledge and Innovation Systems (AKIS) [34], positioning farmer–vet interaction as a locus of innovation where trust, credibility, and decision-making are continually negotiated. Challenges such as antimicrobial use, preventive uptake, or perceptions of veterinary independence can thus be understood as products of multi-level dynamics. For example, a farmer's trust in a vet (individual) may depend on technical knowledge (individual, interpersonal) as well as systemic conditions like financial incentives and competing advice (interprofessional, systemic) —factors shown in 4.1 to structure both collaboration and tension. Importantly, situated learning does not inherently produce more responsible outcomes; rather, it describes how responsibility is negotiated within existing structural incentives and relational dynamics, which may both enable and constrain change.

### 4.3 Limitations

This scoping review has several limitations. First, although we searched multiple databases, the review was limited to English-language publications, which may have excluded relevant studies in other languages. Second, the evidence base is heavily concentrated in European contexts. However, as no geographical restrictions were applied in the search strategy, this concentration reflects the current distribution of published empirical research rather than a regional focus of the review itself. Major pig-producing regions such as Asia and North America remain underrepresented, indicating an important gap in the literature. Furthermore, certain dimensions of veterinary and farmer practice—such as regulatory reporting obligations and the implementation of compulsory disease control programmes—were not prominently examined in the included empirical findings, which centred primarily on advisory and farm-level interaction. Third, the heterogeneity of included studies and the absence of standardized outcome measures constrained our ability to compare findings. Fourth, the inclusion of grey literature was partial, and relevant practitioner insights may not have been captured systematically. Finally, as with all scoping reviews, we did not conduct a formal risk-of-bias assessment, meaning that the strength of evidence cannot be directly inferred.

### 4.4 Recommendations for policy and practice

Building on our findings, several strategic recommendations emerge:

1. *Reform payment models to decouple income from drug sales and reward preventive work.* This recommendation builds on theme 3 subtheme 3, where financial dependencies shaped practice. Aligning financial incentives with preventive care is fundamental to mitigating perceived and actual conflicts of interest that undermine veterinary credibility.
2. *Coordinate advisory roles across professions to reduce contradictory guidance.* This reflects theme 3 subtheme 5, where multiple advisors created confusion. Greater interprofessional coherence is necessary to prevent fragmented decision-making and to restore clarity in farmer–vet collaboration.
3. *Support relationship-building by compensating advisory time, enabling vets to act as partners rather than transactional providers.* This for example links directly to Theme 1's emphasis on trust and credibility. Without structural recognition of advisory work, the relational foundation of trust that underpins effective learning and behavioral change remains fragile.
4. *Institutionalize structured deliberation of trade-offs in herd health decision-making.* This responds to Theme 2's finding that misalignments often reflect differing value priorities rather than knowledge deficits alone. Creating dedicated moments to explicitly discuss these trade-offs, for example through periodic herd health strategy meetings or structured review of antibiotic use, can help surface implicit assumptions before crisis-driven decisions occur. Here, deliberation refers to making implicit negotiation processes explicit, by collectively articulating underlying values, assumptions, and trade-offs that would otherwise remain tacit in day-to-day decision-making. In light of Theme 3's identification of “farm blindness,” mechanisms such as peer consultation or periodic external review (e.g., second opinions) may help maintain critical distance while preserving relational trust. While these might be implemented on some farms already, the review indicates that this is not always the case.
5. *Invest in sector-specific veterinary expertise through continuous professional development, particularly in preventive strategies.* This for example responds to the knowledge gaps highlighted in theme 3 subtheme 4. Strengthening preventive expertise is essential not only for improving herd health outcomes, but for shifting the structural reliance on curative, antibiotic-cantered practices identified in this review.
6. *Redefine veterinary roles around collaboration and shared responsibility, moving beyond unrealistic ideals of “perfect advice.”* This directly addresses theme 3's portrayal of pressures on vets. Recognizing veterinary work as relational and negotiated rather than technocratic helps move beyond unrealistic expectations and strengthens shared accountability for animal health.

### 4.5 Research agenda

Although the analytical framework used in this review was informed in part by dairy-sector research, the empirical literature on farmer–veterinarian interaction remains more extensive in dairy than in intensive pig systems. The patterns identified here also suggest several sector-specific features of pig production, particularly the centrality of antimicrobial governance, the scale and integration of production, and the commercial entanglement of veterinary income with drug sales. Comparative research is therefore needed to examine how sector-specific production logics, regulatory pressures, and advisory traditions shape interaction dynamics across livestock systems. A systematic cross-sector comparison was beyond the scope of this review, but would provide valuable insight into the transferability of these findings.

Future research could operationalize the multi-level model across species, systems, and geographical contexts. Given the strong European concentration of existing studies, comparative research in major pig-producing regions outside Europe is needed to assess the transferability of these findings and to examine how different regulatory, economic, and cultural settings shape farmer–veterinarian interaction. Furthermore, future research could more systematically examine how farming styles and production typologies shape farmer–veterinarian interaction. Variation in attitudes toward health, responsibility, and risk may be partly explained by differing production logics, value orientations, and market positions. While this review focused on farmer–veterinarian interaction, relatively little attention in the included studies was given to intra-professional diversity among veterinarians or to peer-to-peer networks through which advisory norms and practices circulate. Future research could examine how veterinary communities of practice, professional networks, and shared repertoires of action shape interaction with farmers and influence approaches to animal health governance. Moreover, future research could examine how zoonotic risk is (or is not) translated into farm-level advisory interactions, and whether One Health framings meaningfully shape decision-making in intensive pig systems.

The misalignments in Theme 2 suggest that trust should be examined as a multi-level phenomenon spanning individual rapport, interprofessional competition, and systemic incentives. Garforth et al. [55] and Fortané et al. [57] for example show how differing framings of trust and responsibility shape antimicrobial use practices. The systemic pressures highlighted in Theme 3 call for studies of how payment models; regulation; and market structures interact with advisory practice. Studies by Ducrot et al. [59] and Rojo-Gimeno et al. [70] for example illustrate how financial and institutional arrangements shape veterinary decision-making and advisory independence. Furthermore; longitudinal and ethnographic research could help trace how structural pressures interact with everyday situated learning. Finally; building on AKIS perspectives [34]; comparative studies could test how sector-specific conditions shape advisory coherence and responsibility-sharing in light of the transition to resilient livestock farming in terms of farm sustainability and one health challenges. [[15], [25], [38]].

## CRediT authorship contribution statement

**Rolien Willmes:** Writing – review & editing, Writing – original draft, Visualization, Validation, Software, Resources, Project administration, Methodology, Investigation, Funding acquisition, Formal analysis, Data curation, Conceptualization. **Jasper de Vries:** Supervision, Conceptualization, Writing – review & editing. **Bob Mulder:** Supervision, Conceptualization, Writing – review & editing. **P. Marijn Poortvliet:** Conceptualization, Writing – review & editing. **Laurens Klerkx:** Supervision, Conceptualization, Writing – review & editing.

## Declaration of Generative AI and AI-assisted technologies in the writing process

During the preparation of this work, the author used OpenAI's ChatGPT to assist with language editing and clarity improvements. After using this tool, the author reviewed and edited the content as needed and takes full responsibility for the content of the published article.

## Funding

This work was conducted as part of the TKI project “Duurzame verlaging antibioticumgebruik varkenshouderij door collectieve aanpak bedrijfsgebonden dierziekten te beginnen bij PRRSv,” which receives financial support from the Dutch Topsector Agri & Food through a public–private partnership involving Wageningen University and various industry partners. The contribution of Laurens Klerkx was supported by the Agencia Nacional de Investigación y Desarollo, grant ANID CA2FST CIN250015.

The funders had no role in the design, data collection, analysis, interpretation, or writing of this review. The authors are fully responsible for the conduct and reporting of the study.

## Declaration of competing interest

The authors declare no conflict of interest.

## Data Availability

literature review

## Appendix A Supplementary data

Supplementary material 1 Extraction table of selected studies

Supplementary material 2 Search queries

Supplementary material 3 PRISMA-ScR checklist

Supplementary material 4 References selected studies
