# Supplementary material for: Farmer–veterinarian interaction as multi-level situated learning: Negotiating health, risk, and responsibility in intensive pig farming — a scoping review
Source: One Health. 2026 Apr 1;22:101401. doi: 10.1016/j.onehlt.2026.101401 (PMC13091025; doi:10.1016/j.onehlt.2026.101401)
Supplement: Supplementary material 2 — Search queries [file mmc2.docx]

**Web of Science and Scopus:**

Focus on veterinarian involvement

( TITLE-ABS-KEY ( "veterinary practitioner"  OR  "veterinary advice"  OR  "veterinary practice"  OR  "veterinary consultation"  OR  "veterinary surgeon"  OR  veterinarian OR "veterinary service" OR vet ) )  **AND**

Focus on farmer-vet communication

( TITLE-ABS-KEY ( communication OR  communicate  OR  interaction  OR  vet-farmer  OR  farmer-vet  OR veterinarian-farmer OR farmer-veterinarian OR collaboration  OR  co-production OR co-produce  OR  relationship  OR  "relationship factor"  OR  "cultural script" OR  "behavior change"  OR “behaviour change” OR advice  OR  advisory  OR  coach OR coaching  OR  counseling  OR  consult  OR  consultation  OR  extension  OR  "knowledge transfer" OR trust OR management OR program OR protocol) )  **AND**

Focus on PIGS

( TITLE-ABS-KEY ( pig OR piglet  OR sow OR  weaner  OR  "fattening pig"  OR  hog  OR  swine OR porcine ) )

Combined string:

(("veterinary practitioner"  OR  "veterinary advice"  OR  "veterinary practice"  OR  "veterinary consultation"  OR  "veterinary surgeon"  OR  veterinarian OR "veterinary service" OR vet) AND (communication OR  communicate  OR  interaction  OR  vet-farmer  OR  farmer-vet  OR veterinarian-farmer OR farmer-veterinarian OR collaboration  OR  co-production OR co-produce  OR  relationship  OR  "relationship factor"  OR  "cultural script" OR  "behavior change"  OR “behaviour change” OR advice  OR  advisory  OR  coach OR coaching  OR  counseling  OR  consult  OR  consultation  OR  extension  OR  "knowledge transfer" OR trust OR management OR program OR protocol) AND ( pig OR piglet  OR sow OR  weaner  OR  "fattening pig"  OR  hog  OR  swine OR porcine))

**OVID (Agricola and CAB abstracts):**

Combined string:

((veterinary practitioner?  OR  veterinary advice  OR  veterinary practice?  OR  veterinary consultation?  OR  veterinary surgeon?  OR  veterinarian OR veterinary service? OR vet?) AND (communication? OR  communicate?  OR  interaction?  OR  vet?-farmer?  OR  farmer?-vet?  OR veterinarian?-farmer? OR farmer?-veterinarian? OR collaboration?  OR  co-production? OR co-produce?  OR  relationship?  OR  relationship factor?  OR  cultural script? OR  behavior change  OR behaviour change OR advice  OR  advisory  OR  coach OR coaches OR coaching  OR  counseling  OR  consult?  OR  consultation?  OR  extension  OR  knowledge transfer OR trust OR management OR program? OR protocol?) AND ( pig? OR piglet?  OR sow? OR  weaner?  OR  fattening pig?  OR  hog?  OR  swine OR porcine))
