# Supplementary material for: Farmer–veterinarian interaction as multi-level situated learning: Negotiating health, risk, and responsibility in intensive pig farming — a scoping review
Source: One Health. 2026 Apr 1;22:101401. doi: 10.1016/j.onehlt.2026.101401 (PMC13091025; doi:10.1016/j.onehlt.2026.101401)
Supplement: Supplementary material 4 — References selected studies [file mmc4.docx]

Adebowale, O. O., Adeyemo, F. A., Bankole, N., Olasoju, M., Adesokan, H. K., Fasanmi, O., Adeyemo, O., Awoyomi, O., Kehinde, O., & Fasina, F. O. (2020). Farmers’ perceptions and drivers of antimicrobial use and abuse in commercial pig production, Ogun State, Nigeria. *International Journal of Environmental Research and Public Health, 17*(10), 3579. https://doi.org/10.3390/ijerph17103579

Alarcon, P., Wieland, B., Mateus, A. L. P., & Dewberry, C. (2014). Pig farmers’ perceptions, attitudes, influences and management of information in the decision-making process for disease control. *Preventive Veterinary Medicine, 116*(3–4), 223–242.

Alawneh, J. I., Barnes, T. S., Parke, C., Lapuz, E., David, E., Basinang, V., Baluyut, A., Villar, E., Lopez, E. L., & Blackall, P. J. (2014). Description of the pig production systems, biosecurity practices and herd health providers in two provinces with high swine density in the Philippines. *Preventive Veterinary Medicine, 114*(2), 73–87.

Backhans, A., Sjölund, M., Lindberg, A., & Emanuelson, U. (2016). Antimicrobial use in Swedish farrow-to-finish pig herds is related to farmer characteristics. *Porcine Health Management, 2*, 18.

Ball, B., & de Lange, C. F. M. (2014). Participants at swine knowledge transfer events and their information preferences. In *Proceedings of the 33rd Annual Centralia Swine Research Update* (Kirkton-Woodham Community Centre, Ontario, Canada).

Bíró, O., Özsvári, L., & Lakner, Z. (2006). The veterinary medicine in upgrading of competitiveness of Hungarian pig-production sector. *Acta Agraria Kaposváriensis, 10*(2), 109–117.

Coyne, L. A., Pinchbeck, G. L., Williams, N. J., Smith, R. F., Dawson, S., Pearson, R. B., & Latham, S. M. (2014). Understanding antimicrobial use and prescribing behaviours by pig veterinary surgeons and farmers: A qualitative study. *Veterinary Record, 175*(23), 593.

Coyne, L. A., Latham, S. M., Williams, N. J., Dawson, S., Donald, I. J., Pearson, R. B., Smith, R. F., & Pinchbeck, G. L. (2016). Understanding the culture of antimicrobial prescribing in agriculture: A qualitative study of UK pig veterinary surgeons. *Journal of Antimicrobial Chemotherapy, 71*(11), 3300–3312.

Coyne, L. A., Latham, S. M., Dawson, S., Donald, I. J., Pearson, R. B., Smith, R. F., Williams, N. J., & Pinchbeck, G. L. (2019). Exploring perspectives on antimicrobial use in livestock: A mixed-methods study of UK pig farmers. *Frontiers in Veterinary Science, 6*, 257.

de Groot, J., Eijck, I. A. J. M., & Boersma, W. J. A. (2004). Vaccinations in pig health care in the Netherlands: Analysis of a questionnaire among veterinarians. *Tijdschrift voor Diergeneeskunde, 129*(8), 252–260.

Diana, A., Snijders, S., Rieple, A., & Boyle, L. (2021). Why do Irish pig farmers use medications? Barriers for effective reduction of antimicrobials in Irish pig production. *Irish Veterinary Journal, 74*, 7.

Ducrot, C., Guenin, M. J., Hémonic, A., Rousset, N., Carré, Y., Facon, C., Le Coz, P., Marguerie, J., Petiot, J. M., Jarnoux, M., Leblanc-Maridor, M., Paul, M., Molia, S., & Belloc, C. (2022). Towards a better use of antimicrobials on farms: Insights from a participatory approach in the French pig and poultry sectors. *Antibiotics, 11*(4), 470.

Dupont, N., Diness, L. H., Fertner, M., Kristensen, C. S., & Stege, H. (2017). Antimicrobial reduction measures applied in Danish pig herds following the introduction of the “Yellow Card” antimicrobial scheme. *Preventive Veterinary Medicine, 138*, 9–16.

Eriksen, E. O., Pedersen, K. S., Larsen, I., & Nielsen, J. P. (2022). Evidence-based recommendations for herd health management of porcine post-weaning diarrhea. *Animals, 12*(3), 300.

Fortané, N., Bonn-Beteaugr, F., Hémonic, A., Samedi, C., Savy, A., & Belloc, C. (2015). Learning processes and trajectories for the reduction of antibiotic use in pig farming: A qualitative approach. *Antibiotics, 4*(4), 497–513.

Garforth, C. J., Bailey, A. P., & Tranter, R. B. (2013). Farmers’ attitudes to disease risk management in England: A comparative analysis of sheep and pig farmers. *Preventive Veterinary Medicine, 110*(3–4), 456–466.

Giersberg, M. F., & Meijboom, F. L. B. (2023). As if you were hiring a new employee: On pig veterinarians’ perceptions of professional roles and relationships in the context of smart sensing technologies in pig husbandry in the Netherlands and Germany. *Agriculture and Human Values.*

Golding, S. E., Ogden, J., & Higgins, H. M. (2019). Shared goals, different barriers: A qualitative study of UK veterinarians’ and farmers’ beliefs about antimicrobial resistance and stewardship. *Frontiers in Veterinary Science, 6*, 132.

Hallenberg, G. S., Jiwakanon, J., Angkititrakul, S., Kang-Air, S., Osbjer, K., Lunha, K., Sunde, M., Järhult, J. D., van Boeckel, T. P., Rich, K. M., & Magnusson, U. (2020). Antibiotic use in pig farms at different levels of intensification—Farmers’ practices in northeastern Thailand. *PLoS ONE, 15*(2), e0228597.

Hinchliffe, S., & Ward, K. J. (2014). Geographies of folded life: How immunity reframes biosecurity. *Geoforum, 53*, 136–144.

Ison, S. H., & Rutherford, K. M. D. (2014). Attitudes of farmers and veterinarians towards pain and the use of pain relief in pigs. *The Veterinary Journal, 202*(3), 622–627.

Joly, N., Adam, C., Bonnet-Beaugrand, F., Defois, J., Ducrot, C., Fortané, N., Frappat, B., Gros, A., Hellec, F., Manoli, C., Paul, M., Poizat, A., & Samedi, C. (2016). Experiments in animal farming practice: The case of decreasing the use of antimicrobials in livestock (France). In *Proceedings of the 12th European IFSA Symposium*.

Kongsted, H., & Loughlin, E. T. M. (2023). Lowering antibiotic usage and phasing out pharmaceutical zinc oxide in Danish pig herds: Pig farmers’ and veterinarians’ experiences and perceptions. *Livestock Science, 266*, 105119.

Laanen, M., Maes, D., Hendriksen, C., Gelaude, P., De Vliegher, S., Rosseel, Y., & Dewulf, J. (2014). Pig, cattle and poultry farmers with a known interest in research have comparable perspectives on disease prevention and on-farm biosecurity. *Preventive Veterinary Medicine, 115*(1–2), 1–9.

Lekagul, A., Tangcharoensathien, V., Mills, A., Rushton, J., & Yeung, S. M. (2020). How antibiotics are used in pig farming: A mixed-methods study of pig farmers, feed mills and veterinarians in Thailand. *BMJ Global Health, 5*(2), e001918.

Maes, D., Vander Beken, H., Dewulf, J., De Vliegher, S., Castryck, F., & De Kruif, A. (2010). The functioning of the veterinarian in the Belgian pig sector: A questionnaire survey of pig practitioners. *Vlaams Diergeneeskundig Tijdschrift, 79*(2), 90–96.

Marier, E., Smith, P., Ellis-Iversen, J., Watson, E., Armstrong, D., Hogeveen, H., & Cook, A. J. C. (2016). Changes in perceptions and motivators that influence the implementation of on-farm Salmonella control measures by pig farmers in England. *Preventive Veterinary Medicine, 133*, 107–116.

Mercy, A. (1991). Veterinary consultancy boosts piggery profits. *Journal of Agriculture, Western Australia, 32*(4), 124–127.

Noremark, M., Lewerin, S. S., Ernholm, L., & Frössling, J. (2016). Swedish farmers’ opinions about biosecurity and their intention to make professionals use clean protective clothing when entering the stable. *Frontiers in Veterinary Science, 3*, 46.

Özsvári, L., Bíró, O., & Lakner, Z. (2012). Role of veterinary management in increasing pig breeding efficiency: A methodological approach. *Studies in Agricultural Economics, 114*(1), 34–41.

Rojo-Gimeno, C., Dewulf, J., Maes, D., & Wauters, E. (2018). A systemic integrative framework to describe comprehensively a swine health system: Flanders as an example. *Preventive Veterinary Medicine, 158*, 103–112.

Rosanowski, S. M., Magouras, I., Ho, W.-C., Yiu, W. C. J., Pfeiffer, D. U., & Zeeh, F. (2023). The challenges of pig farming in Hong Kong: A study of farmers’ perceptions and attitudes towards a pig health and production management service. *BMC Veterinary Research, 19*, 6.

Simon-Grifé, M., Martín-Valls, G. E., Vilar-Ares, M. J., García-Bocanegra, I., Martín, M., Mateu, E., & Casal, J. (2013). Biosecurity practices in Spanish pig herds: Perceptions of farmers and veterinarians of the most important biosecurity measures. *Preventive Veterinary Medicine, 110*(3–4), 223–231.

Speksnijder, D. C., Jaarsma, A. D. C., van der Gugten, A. C., Verheij, T. J. M., & Wagenaar, J. A. (2015). Determinants associated with veterinary antimicrobial prescribing in farm animals in the Netherlands: A qualitative study. *Zoonoses and Public Health, 62*(Suppl. 1), 39–51.

Teixeira-Costa, C., Andraud, M., Rose, N., & Salines, M. (2020). Controlling hepatitis E virus in the pig production sector: Assessment of the technical and behavioural feasibility of on-farm risk mitigation strategies. *Preventive Veterinary Medicine, 182*, 105103.

Theodoropoulos, G., Stevens, K. B., Hartsa, A., Theodoropoulou, H., & Pfeiffer, D. U. (2009). Farm-level factors associated with above-average production on pig farms in Evia, Greece. *Preventive Veterinary Medicine, 89*(3–4), 233–240.

van Asseldonk, M., de Lauwere, C., Bonestroo, J., Bondt, N., & Bergevoet, R. (2020). Antibiotics use versus profitability on sow farms in the Netherlands. *Preventive Veterinary Medicine, 182*, 105101.

Visschers, V. H. M., Iten, D. M., Riklin, A., Hartmann, S., Sidler, X., & Siegrist, M. (2014). Swiss pig farmers’ perception and usage of antibiotics during the fattening period. *Livestock Science, 162*, 223–232.

Visschers, V. H. M., Backhans, A., Collineau, L., Loesken, S., Nielsen, E. O., Postma, M., Belloc, C., Dewulf, J., Emanuelson, U., Grosse Beilage, E., Siegrist, M., Sjölund, M., & Stark, K. D. C. (2016). A comparison of pig farmers’ and veterinarians’ perceptions and intentions to reduce antimicrobial usage in six European countries. *Zoonoses and Public Health, 63*(7), 534–547.

Wise, J. K. (1988). Livestock producers’ ratings of alternative veterinary information sources. *Journal of the American Veterinary Medical Association, 192*(5), 607–610.
